# Progression of Amplitude-Integrated Electroencephalography and Neurological Outcomes in Neonates With Hypoxic-Ischemic Encephalopathy: A Single-Institution Cohort Study in Vietnam

**DOI:** 10.7759/cureus.62317

**Published:** 2024-06-13

**Authors:** Thu Hua, Thien T Nguyen, Tinh T Nguyen

**Affiliations:** 1 Department of Pediatrics, Children's Hospital 2, Ho Chi Minh, VNM; 2 Department of Pediatrics and Neonatology, Children's Hospital 2, Ho Chi Minh, VNM; 3 Department of Pediatrics, University of Medicine and Pharmacy at Ho Chi Minh City, Ho Chi Minh, VNM; 4 Department of Neonatology, Children's Hospital 2, Ho Chi Minh, VNM; 5 Department of Neonatology, Ho Chi Minh University Medical Center, Ho Chi Minh, VNM

**Keywords:** seizure, neonate, hypothermia, aeeg, hypoxic-ischemic encephalopathy

## Abstract

Background

The characteristics of amplitude-integrated electroencephalography (aEEG) are associated with neurological outcomes in neonates with hypoxic-ischemic encephalopathy (HIE). We perform a longitudinal analysis of continuous monitoring of aEEG during therapeutic hypothermia and explore the association between aEEG interpretation and clinical neurological outcomes.

Method

We conducted a prospective cohort study on HIE neonates undergoing hypothermia with aEEG monitoring.

Results

A total of 37 HIE infants underwent hypothermia with improved aEEG background activity in 28 (75.7%) neonates, of which 18 (48.6%) neonates had background activity returned to a continuous pattern, and the median recovery time was 26.5 hours. Sleep-wake cycle (SWC) appeared in 14 (37.8%) cases, with a median onset time of 34.5 hours. Seizure activity on aEEG was present in 26 (70.3%) infants. Factors associated with poor outcomes at discharge included low voltage or flat trace background activity, a lack of improvement in background activity after hypothermia, and the absence of SWC. Neonates who took longer than 62 hours to return to continuous background activity (time to normal trace) or did not have SWC before the end of hypothermia were more likely to have unfavorable outcomes at discharge.

Conclusions

Longitudinal analysis of aEEG during hypothermia should be implemented in neonatal care units. The progression of these features on aEEG may predict neurological outcomes for HIE neonates.

## Introduction

Hypoxic-ischemic encephalopathy (HIE) is a consequence of perinatal asphyxia, causing approximately one million deaths annually worldwide, with 99% occurring in low- and middle-income countries [1]. Moderate to severe HIE can lead to death or long-term neurological impairment of up to 40-50% [2].

Several tools have been researched, developed, and compared to accurately predict neurological outcomes in HIE cases, including clinical examination scoring, neuroimaging, and electroencephalography studies. Among them, amplitude-integrated electroencephalography (aEEG) is a useful and convenient bedside tool for continuous monitoring of brain function, commonly used in various brain injury conditions, including HIE. Interpretation and classification of aEEG are based on background activity patterns, sleep-wake cycles (SWCs), and seizure patterns [3]. In high-income countries, many studies have demonstrated the role of aEEG monitoring in predicting neurological outcomes and raised questions about interpreting the significance of aEEG at different time points and in progression [4-6]. However, in low- and middle-income countries, studies have shown that hypothermia therapy does not improve neurological outcomes, including mortality and disabilities, which could be explained by the difference in population comorbidities and socioeconomic risk factors, as well as limited healthcare resources in general and healthcare in intensive care units in particular. Additionally, data on aEEG in HIE in low- and middle-income countries are still incomplete, making it difficult to integrate aEEG into clinical practice. However, in low- and middle-income countries, data on aEEG monitoring are still scarce, challenging the integration of aEEG into clinical practice.

This study aims to investigate the progression of aEEG during hypothermia and explore the association between aEEG interpretation and clinical neurological outcomes.

## Materials and methods

Ethics approval

The study received ethical approval from the Ethics Committee of Children’s Hospital 2 on May 27, 2022 (approval number 03/23-BVND2).

Study design

This prospective cohort study was conducted at Children's Hospital 2, Ho Chi Minh, Vietnam, for 13 months, from June 1, 2022 to June 30, 2023.

Setting

This study included all neonates diagnosed with HIE undergoing hypothermia with aEEG monitoring at the NICU of Children’s Hospital 2, a tertiary neonatal unit in Southern Vietnam that has been applying hypothermia and continuous aEEG monitoring for many years.

Participants

Inclusion Criteria

All neonates who met the following criteria were included in the study: gestational age over 35 weeks, birth weight over 1,800 grams, admission within six hours of birth, and diagnosis of HIE under the following conditions: (i) evidence of acidosis with umbilical cord blood gas result of pH ≤7.0 or base deficit ≥16, or (ii) perinatal asphyxia with resuscitation lasting more than 10 minutes or APGAR score ≤5 at 10 minutes, and (iii) moderate to severe neonatal encephalopathy according to SARNAT staging requiring hypothermia and aEEG monitoring for at least 72 hours [7].

Exclusion Criteria

All neonates who met any of the following criteria were excluded from the study: severe intrauterine growth restriction; chromosomal abnormalities or severe congenital multiorgan defects; congenital brain abnormalities; severe traumatic brain injury or intracranial hemorrhage; and congenital disorders related to severe thrombocytopenia or coagulation disorders.

Variables

The dependent variables were the characteristics of aEEG, including background activity patterns categorized according to Hellstrom-Westas classification into five types [3]: continuous normal voltage (CNV), discontinuous normal voltage, burst suppression (BS), low voltage (LV), and flat trace (FT). Severe abnormal patterns include BS, LV, and FT (see Appendix).

Five time points of assessment were measured from the start of hypothermia: T0 (start of hypothermia); T1 (0-12 hours); T2 (12-24 hours); T3 (24-48 hours); T4 (48-72 hours); and T5: rewarming phase (72-78 hours). The progression of background activity includes two types: improved when the background activity at T5 becomes better than at T0, not improved when the background activity at T5 becomes worse than at T0, or similar to T0. Additionally, time to normalization of background activity was measured, including time to normal trace (TTNT) and time to appearance of SWCs - time to SWC (TSWC).

The independent variables were the outcomes at discharge, including death, major neurological morbidities (one of the following: seizure requiring anticonvulsants, nasogastric tube feeding, increased or decreased muscle tone, or respiratory support dependence), and recovery (a normal neurological exam by an experienced neonatologist in charge).

Study protocol

Screening of HIE-diagnosed neonates treated at the NICU of Children’s Hospital 2 from June 1, 2022, to June 30, 2023, involved screening through the inclusion and exclusion criteria. Hypothermia treatment using CritiCool PRO (Belmont Medical Technologies, Billerica, United States) for 72 hours with clinical and continuous aEEG monitoring was administered, with monitoring continuing until discharge. Epidemiological, obstetric, clinical, laboratory results, MRI, aEEG, and treatment data were collected. aEEG activity was recorded in the cooling machine, and an experienced neonatologist in charge assessed the traces to classify the patterns. The worst pattern recorded in a time frame would be collected from the data set as the pattern of that time frame.

The hypothermia device used is the Criticool PRO integrated with the VitaLogik Patient Monitor (Mennen Medical, Yavne, Israel). The monitor is an EEG amplifier and aEEG, which has two channels of EEG/aEEG and can provide a monitor for vital signs, electrocardiography, and aEEG. The EEG and aEEG are displayed and stored simultaneously with the patient’s vital signs. EEG/aEEG and vital signs can be downloaded to a PC for display and analysis.

Brain MRI was performed within the first two weeks at the Imaging Department of Children’s Hospital 2 using Philips Multiva 1.5T (Philips, Amsterdam, The Netherlands). The imaging process includes MRI sequences: axial T2-weighted, diffusion + apparent diffusion coefficient map, susceptibility-weighted imaging; coronal flair, sagittal T1-weighted, and T1-weighted 3D. The MRI result was assessed by the radiologist in charge of the Imaging Department, and due to limitations in the setting, MRI scoring was not applied during interpretation.

Statistical analysis

Data was encoded, entered, and managed using MS Excel (Microsoft Corporation, Redmond, United States). Data processing and statistical analysis were performed using IBM SPSS Statistics for Windows, Version 20.0 (Released 2011; IBM Corp., Armonk, NY, USA). Results are presented in tables or graphs. Categorical variables are compared using the chi-square test, Fisher’s exact test, or McNemar’s test. Continuous variables were compared using a t-test. The relationship between independent variables (quantitative) and dependent variables (categorical) is assessed using the ANOVA test. Receiver operating characteristic (ROC) curve analysis is conducted to determine the cutoff thresholds, sensitivity, and specificity of TTNT and TSWC for predicting neurological outcomes.

## Results

From June 2022 to June 2023, a total of 37 neonates met the inclusion criteria, with no cases excluded from the study.

The male-to-female ratio was 56.8%, with an average gestational age of 38.9 weeks and an average birth weight of 3096 grams. Vaginal deliveries accounted for 56.8%, with all cases being born in healthcare facilities. The average age at the start of hypothermia therapy was 4.2 hours. Five cases did not undergo endotracheal intubation during resuscitation but were later intubated before transfer to warrant inter-hospital transport. The median resuscitation time was 10 minutes. Our study had 34 cases (91.9%) undergoing brain MRI, with a median age at the time of MRI being seven days, ranging from four to 22 days. Three cases did not undergo MRI due to parental refusal. Among the 28 cases with observed abnormalities within the brain tissue signal consistent with HIE injury, 17 cases (60.7%) had watershed area abnormalities, 22 cases (78.5%) had basal ganglia/thalamus abnormalities, nine cases (32.1%) had posterior limb of the internal capsule (PLIC) abnormalities, five cases (17.8%) had brainstem abnormalities, 16 cases had cortical abnormalities (57.1%), and 20 cases had subcortical white matter signal abnormalities (71.4%). Brain hemorrhage was present in 14 cases (50%) (Table 1).

**Table 1:** Characteristics of the study sample (n = 37) Data are presented as n (%), mean (SD), or median (IQR). * Multiple lesions can be observed on brain MRI in neonates. BGT, basal ganglia/thalamus; PLIC, posterior limb of the internal capsule

Characteristics	Results
Male	21 (56.7)
Gestational age (weeks)	38.9 ± 1.1
Birth weight (grams)	3,096 ± 679
Age at hypothermia (hours)	4.2 ± 1.1
Vaginal birth	21 (56.8)
Delivery room resuscitation (n = 35)	
Positive pressure ventilation via mask	35 (100)
Chest compression	25 (71.4)
Intubation	25 (71.4)
Adrenalin	17 (48.5)
Resuscitation time (minutes), median (IQR)	10 (5.0; 15.0)
APGAR score	
One minute (n = 37)	3.4 ± 1.4
Five minutes (n = 35)	5.0 ± 1.5
Ten minutes (n = 12)	5.7 ± 2
Blood gas during one hour of age (n = 17)	
pH	7.02 ± 0.13
Base excess (mmol/L)	-20.5 ± 3.36
Seizure on admission	22 (59.5)
SARNAT classification	
Grade I	1 (2.7)
Grade II	35 (94.6)
Grade III	1 (2.7)
Invasive ventilation support	28 (75.7)
Invasive ventilation support duration (days)	5 (1.0; 8.0)
Vasopressor support	11 (29.7)
Brain MRI (n = 34)*	
Watershed area	17 (60.7)
BGT	22 (78.5)
PLIC	9 (32.1)
Brain stem	5 (17.8)
Cortex	16 (57.1)
Subcortical white matter	20 (71.4)
Brain hemorrhage	14 (50.0)

At discharge, there were seven deaths (18.9%), all of which were due to severe neurological conditions with invasive respiratory support dependence; 12 cases (32.4%) recovered completely; and 18 cases (48.6%) had major neurological morbidities, including five cases with seizures requiring anticonvulsants (13.5%), three cases requiring nasogastric tube feeding (8.1%), 18 cases with increased or decreased muscle tone (48.6%), and no cases requiring respiratory support. The average length of treatment in the NICU was 8.3 days, and the average length of hospital stay was 21.6 days (Table 2).

**Table 2:** Outcomes at discharge (n = 37) Data are presented as n (%).

Outcomes	Results
Death	7 (19.0)
Major neurological morbidities	18 (48.6)
Seizures requiring anticonvulsants	5 (13.5)
Nasogastric tube feeding	3 (8.1)
Increase or decrease in muscle tone	18 (48.6)
Respiratory support dependence	0 (0.0)
Recovery	12 (32.4)

**Figure 1:**
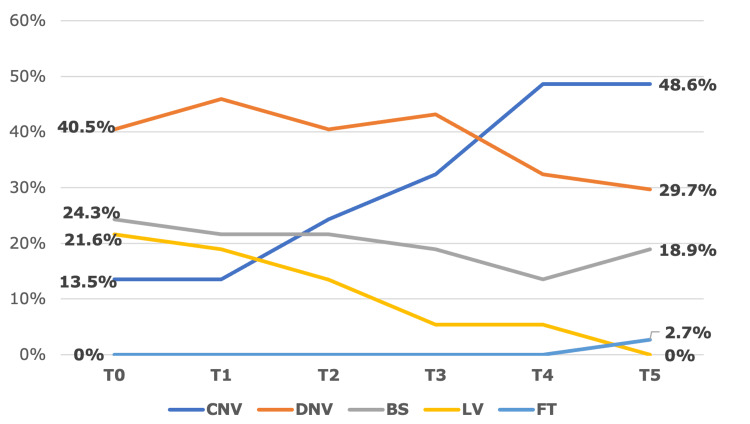
Background activity progression on aEEG The pattern of background activity during hypothermia improved over time, with severe activity (BS, LV, and FT pattern) decreasing from 46% at T0 to 21.6% at T5 (p = 0.03). Statistically significant at p < 0.05. From the start of hypothermia: T0 (start); T1 (0-12 hours); T2 (12-24 hours); T3 (24-48 hours); T4 (48-72 hours); and T5 - rewarming phase (72-78 hours) aEEG, amplitude-integrated electroencephalography; BS, burst suppression; CNV, continuous normal voltage; DNV, discontinuous normal voltage; FT, flat trace; LV, low voltage

In our study, the overall background activity on aEEG during hypothermia therapy improved over time, with the severe abnormal patterns (BS, LV, and FT) decreasing from 17 cases (46%) at T0 to eight cases (21.6%) at T5 (p = 0.03) (Figure 1).

**Figure 2:**
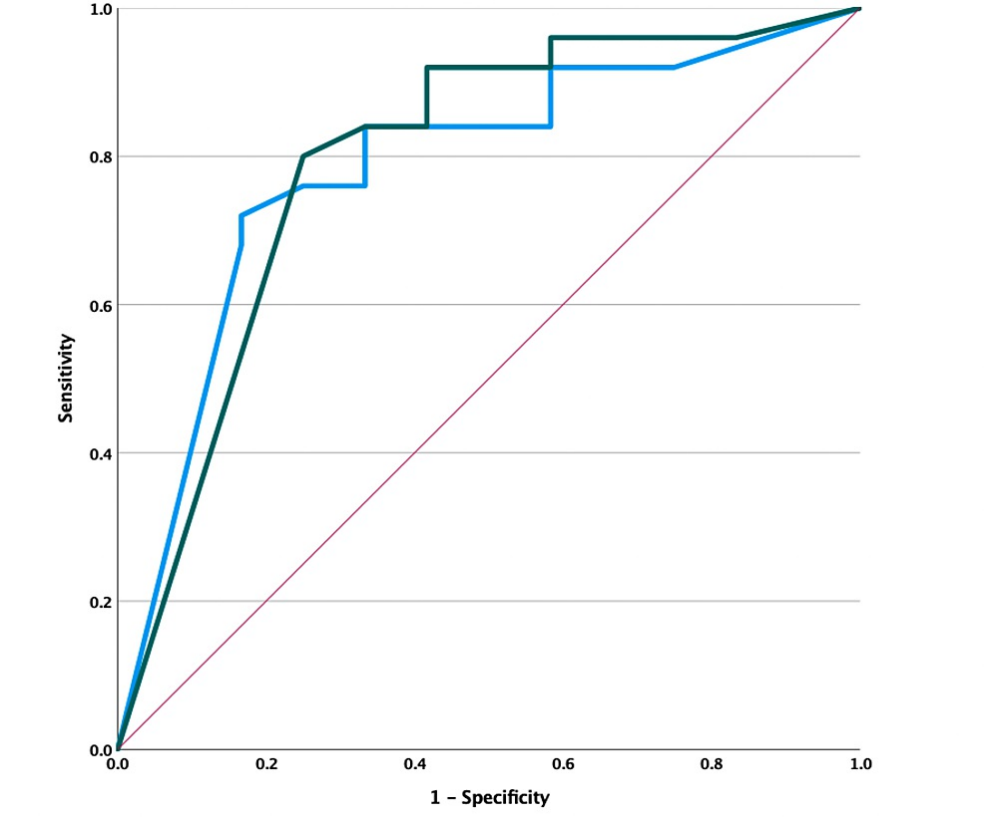
ROC curve illustrating TTNT and TSWC to predict outcomes at discharge The cutoff threshold of TTNT was 62 hours, with AUC = 0.785, sensitivity 72%, specificity 83.3%, LR+ 4.3, and p = 0.006. The cutoff threshold of TSWC was 84.5 hours, with AUC = 0.795, sensitivity 80%, specificity 75%, LR+ 3.2, and p = 0.004. Statistically significant at p < 0.05. Note: blue line: TTNT; green line: TSWC; red line: reference line ROC, receiver operating characteristic; TSWC, time to sleep-wake cycle; TTNT, time to normal trace

All eight cases with LV or FT patterns recorded at any time point had adverse outcomes (either death or major neurological morbidities) at discharge (p = 0.027, chi-square test). There were five cases with CNV patterns from the onset of hypothermia, and all of them had good outcomes at discharge. All nine cases with no improvement in background activity after hypothermia had poor outcomes at discharge (including six deaths and three with major neurological morbidities), and the difference was statistically significant with p < 0.001 compared to the group with improved background activity after hypothermia. Background activity patterns on aEEG at T3, T4, and T5 (which corresponded to the period from 48 hours to the end of rewarming) were associated with outcome at discharge with p < 0.05. Our study identified 18 cases with patterns returning to normal trace, with a median TTNT of 26.5 hours. The ROC curve identified the threshold of TTNT at the 62-hour cutoff, with an area under the curve (AUC) of 0.785, sensitivity of 72%, specificity of 83.3%, positive likelihood ratio (LR+) of 4.3, and p = 0.006. There were 14 cases with SWC that appeared during hypothermia in our study (37.8%), of which nine had a full recovery and five had major neurological morbidities at discharge, with a median TSWC of 34.5 hours. The presence of SWC was significantly related to discharge outcome (p = 0.002). Cases with no SWC until the end of hypothermia were more likely to have adverse outcomes at discharge, with an AUC of 0.795, a sensitivity of 80%, a specificity of 75%, an LR+ of 3.2, and a p-value of 0.004 (Figure 2).

Our study had 11 cases (29.7%) with no recorded seizures on EEG. The group with seizure activity included 26 infants, with repetitive seizures in 17 (45.9%) and status epilepticus in nine (24.3%). The number of cases with aEEG-detected seizure was higher than those observed clinically (26 vs. 22 cases, respectively) (Table 3).

**Table 3:** aEEG characteristics (n = 37) Data are presented as n (%) or median (IQR). From the start of hypothermia: T0 (start); T1 (0-12 hours); T2 (12-24 hours); T3 (24-48 hours); T4 (48-72 hours); and T5 - rewarming phase (72-78 hours) aEEG, amplitude-integrated electroencephalography; BS, burst suppression; CNV, continuous normal voltage; DNV, discontinuous normal voltage; FT, flat trace pattern; LV, low voltage; SWC, sleep-wake cycle; TSWC, time to sleep-wake cycle; TTNT, time to normal trace

aEEG characteristics	Results
Severe abnormal pattern	
At T0	17 (46.0)
At T5	08 (21.6)
Pattern not improved	09 (24.3)
TTNT (hours) (n = 18)	26.5 (0; 50)
Absent SWC	23 (62.2)
TSWC (hours) (n = 14)	34.5 (4.5; 58.3)
Seizure activity	26 (70.3)
Onset of seizure activity (hours) (n = 26)	0 (0-9.3)

## Discussion

Predicting neurological outcomes in the HIE population remains an unmet need as several tools are still under validation, including neurophysiological tests like aEEG and imaging techniques. A systematic review, including 26 studies conducted in 1,458 cases of HIE, concluded that the AUC of aEEG analysis was 0.78 and the AUC of brain MRI was 0.88 to predict unfavorable outcomes [8]. In the pre-hypothermia era, aEEG in the first six hours was considered the best short- and mid-term neurological prognostic tool in HIE [9]. Since the widespread implementation of hypothermia therapy, the interpretation of aEEG results in the early hours has been significantly changed due to the neuroprotective effect of hypothermia, which can lead to the normalization of initial abnormal background patterns [4,10,11]. Choosing the appropriate time point to evaluate and integrate relevant factors to interpret aEEG results accurately is crucial in clinical practice.

In our study, neonates with LV or FT background patterns were more likely to have adverse outcomes at discharge (p = 0.027). Merchant and Azzopardi reported that the aEEG characteristic with the best positive predictive value (PPV) for unfavorable neurological outcomes was LV or FT background activity, with PPVs of 59% and 71%, respectively, for the hypothermia and non-hypothermia groups [12]. We identified that background activity patterns on aEEG from 48 hours to the end of rewarming were associated with outcomes at discharge with p < 0.05. International studies have also reported similar results. Ouwehand et al. found that from 36 hours onward, aEEG sensitivity decreased and specificity increased for predicting neurological prognosis, with the highest OR for unfavorable outcomes at 36 hours and the lowest at six hours (101 vs. 9) [4]. Weeke et al. noted that background activity at 36 and 48 hours was strongly associated with neurological development outcomes, with p-values of 0.009 and 0.029, respectively. In contrast, this association was not observed at the onset of hypothermia or after 24 hours. At 36 hours, the OR for abnormal background activity on aEEG to predict poor outcomes was 10.7 (95% CI: 1.9-59.6, p = 0.007) [13]. Meder et al. also confirmed the predictive value of longitudinal analysis of aEEG background with an AUC of 0.90 (95% CI: 0.85-0.95) in an observational study including 149 neonates with HIE [14]. An outcome predictive tool based on the results of this study was built, named the HIE Outcome Prediction using aEEG, HOPE, and is available openly for neonatal institutions. A systematic review conducted by Del Rio et al. identified 17 articles, including data on aEEG in HIE patients treated with and without hypothermia. The study confirmed that among the infants not treated with hypothermia, aEEG background from as early as six hours of birth had a high predictive value (positive post-test probability of 88.2%), while among the infants undergoing hypothermia, aEEG background had a maximum predictive value at 72 hours of birth (positive post-test probability of 95.7%), and the predictive value at six hours was significantly low (59.1%). The study indicated that analysis of the progression of aEEG during hypothermia had improved predictive value in HIE patients [15].

Regarding the improvement in background patterns, TTNT is considered one of the strongest predictors of poor neurological outcomes, with most neonates with favorable outcomes having TTNT within 48 hours. In our study, we identified that the threshold of TTNT was at the 62-hour cutoff, with an AUC of 0.785, sensitivity of 72%, specificity of 83.3%, LR+ of 4.3, and p = 0.006. Our findings were similar to those of international studies. Chandrasekaran et al. conducted a meta-analysis of nine studies involving 520 HIE cases to assess the role of aEEG, reporting the highest OR for adverse outcomes of TTNT was at the 48-hour cutoff (OR 66.9, 95% CI: 19.7-227.2) [9]. Nyman et al. found that among several characteristics of aEEG, the poor recovery of background activity was related to the development of epilepsy at four years of age [16].

The appearance of SWC in term neonates indicates intact brain connectivity and predicts good outcomes in HIE, while the absence of SWC during aEEG monitoring may predict unfavorable outcomes. Thoresen et al. noted the role of time until SWC appearance in predicting neurological outcomes in a study of 74 infants in the CoolCap trial, with a median TSWC of 30 hours, and the absence of SWC was associated with mortality and sequelae, with an OR for adverse outcomes increasing by 1.05 (95% CI: 1.02-1.08, p = 0.02) for every hour of TSWC increase [17]. In recent years, several models have been built to automatically read and analyze basic characteristics on bedside aEEG records during hypothermia, with promising results reported worldwide [18]. The utilization of such a model can support individualized care for HIE patients and aid in early neurological prognosis. Similarly, our study also identified that the presence of SWC was significantly related to discharge outcome (p = 0.002). The ROC curve identified that cases with no SWC until the end of hypothermia were more likely to have adverse outcomes at discharge, with an AUC of 0.795, a sensitivity of 80%, a specificity of 75%, an LR+ of 3.2, and a p-value of 0.004.

In contrast with previous evidence, our study found no association between seizure activity on aEEG and discharge outcome, possibly due to the limited sample size. We estimated that to achieve a statistical power of 80% and a significance level of 0.05, 104 samples in each outcome group would be needed to draw reliable conclusions. In our study, 32 cases received anticonvulsants (phenobarbital), which is higher than the number of cases with seizure activity detected on aEEG and comprised the majority of the study sample. A study investigating the role of aEEG and conventional EEG with video in detecting neonatal seizures confirmed that 42% of cases receiving anti-convulsants did not have seizures on EEG with video [19]. Variane et al. also identified that seizures were more commonly detected on aEEG (at 71.9%) than on clinical observation (at 16.9%) [20]. Although our aEEG monitoring process did not include simultaneous monitoring of sedative-analgesic drug use to assess the duration of drug use and its effects on brain activity, our aEEG data tended to have more prolonged TTNT and TSWC compared to other studies worldwide, possibly due to anticonvulsants. Additionally, many studies also noted that hypothermia therapy could delay the onset of SWC, possibly due to the cold environment limiting sleep regulation [21]. The temperature self-regulation process of the brain is often reduced during deep sleep. Hence, once hypothermia therapy is initiated, the brain will automatically avoid entering deep sleep to reduce the risk of excessive temperature dysregulation, which can be dangerous.

To our knowledge, our study was one of the first to include continuous aEEG monitoring in investigating neurological outcomes in HIE patients in our regions. The study was conducted at the NICU of Children’s Hospital 2, a tertiary neonatal center with many years of experience in implementing hypothermia therapy for HIE cases. Our study had limitations due to the relatively small sample size, which may affect the generalizability of the data. Short-term neurological outcome assessment and its interpretation combined with brain imaging and aEEG may not correlate with long-term neurological prognosis in school-aged children (four to six years old). Therefore, further long-term follow-up studies are needed to validate reliable aEEG interpretation factors for the HIE population.

## Conclusions

Persistent abnormal background activity on aEEG after hypothermia, an LV or FT pattern at any time, TTNT exceeding 62 hours, and the absence of SWC until the end of hypothermia are predictive factors for poor neurological outcomes in HIE neonates. Conversely, cases with improved background activity or CNV patterns at the onset of hypothermia are more likely to have good outcomes. Continuous aEEG monitoring during hypothermia in HIE is feasible, and the progression of these features on aEEG may predict neurological outcomes for HIE infants.

## Figures and Tables

**Figure 3:**
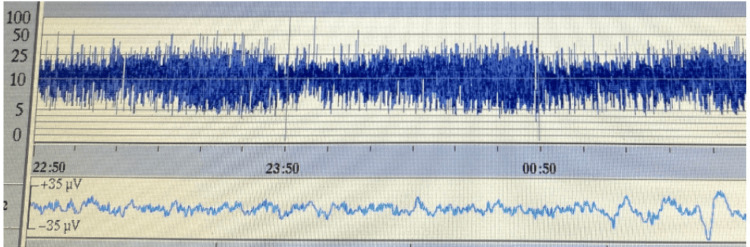
CNV The image was derived from aEEG data records in our study. Waves of continuous pattern; maximum potential >10 μV and minimum potential >5 μV aEEG, amplitude-integrated electroencephalography; CNV, continuous normal voltage

**Figure 4:**
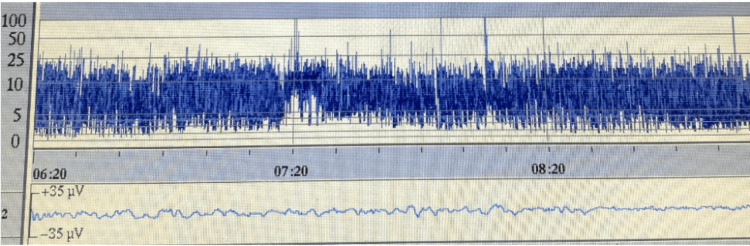
DNV The image was derived from aEEG data records in our study. Discontinuous waves with oscillating minimum potential; maximum potential >10 μV and minimum potential <5 μV aEEG, amplitude-integrated electroencephalography; DNV, discontinuous normal voltage

**Figure 5:**
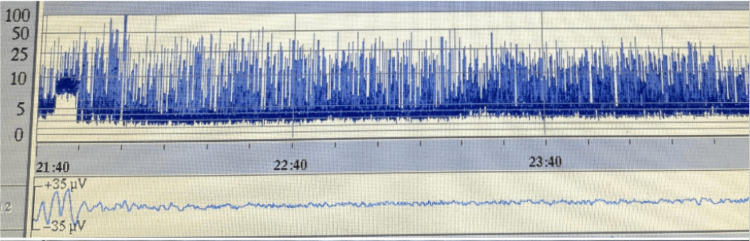
BS The image was derived from aEEG data records in our study. Discontinuous waves with little change in minimum potential and sudden high waves of >25 μV; maximum potential >10 μV and minimum potential <5 μV aEEG, amplitude-integrated electroencephalography; BS, burst suppression

**Figure 6:**
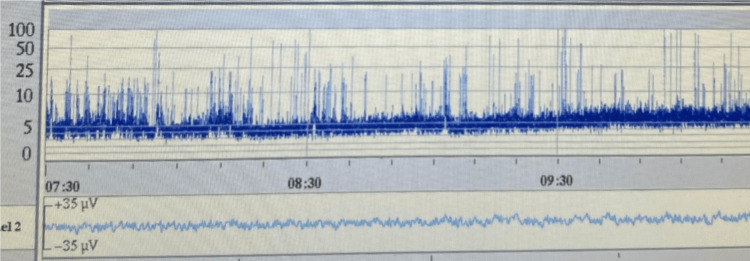
LV The image was derived from aEEG data records in our study. Background waves are continuous with very low potential; maximum potential <10 μV and minimum potential <5 μV aEEG, amplitude-integrated electroencephalography; LV, low voltage

**Figure 7:**
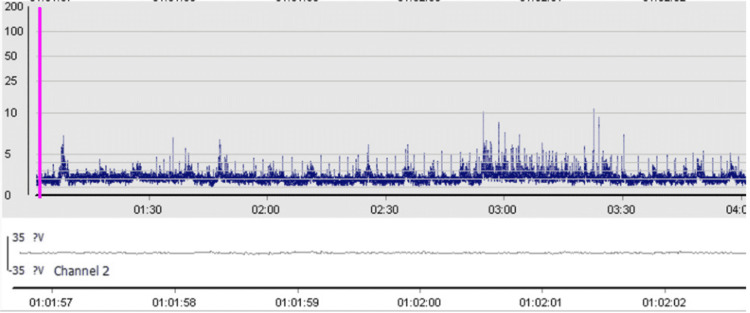
FT The image was derived from aEEG data records in our study. Potential waves are mostly isoelectric with very low potential; maximum potential <5 μV and minimum potential <5 μV aEEG, amplitude-integrated electroencephalography; FT, flat trace

## Appendices

Images of five background activities based on the classification of Hellstrom-Westas and Rosén [3]
